# Single-incision video-assisted thoracic resection for extrapulmonary sequestration: a case report

**DOI:** 10.1186/1749-8090-9-22

**Published:** 2014-01-24

**Authors:** Masaya Tamura, Yosuke Shimizu, Yasuo Hashizume

**Affiliations:** 1 Department of Surgery, Fukui Prefectural Hospital, Yotsui 2-8-1, Fukui, Fukui, 910-8526, Japan

**Keywords:** Extralobar pulmonary sequestration, Video- assisted thoracoscopic surgery, Single- incision thoracoscopic surgery

## Abstract

We describe surgical resection of an extralobar pulmonary sequestration via single-incision thoracoscopic surgery (SITS), which we recommend as a suitable surgical option. A 45-year-old Japanese woman was admitted to our hospital for further examination of chest abnormal shadow. A rigid 5-mm 30° video-thoracoscope, an endograsper and an electric cautery were passed within the same single small incision. The tumor was suspended using articulating endograspers and resected after clipping and ligation of the anomalous vessel. The final pathology was determined an extrapulmonary sequestration.

## Background

Pulmonary sequestration is a rare congenital abnormality of the lower respiratory tract and occurs in 0.15% - 6.4% of all pulmonary malformations [1]. We describe the first case of an extralobar pulmonary sequestration resected via minimally invasive single-incision thoracoscopic surgery (SITS). Furthermore, we discuss the advantages and disadvantages of several practical treatments.

## Case Presentation

**Figure 1:**
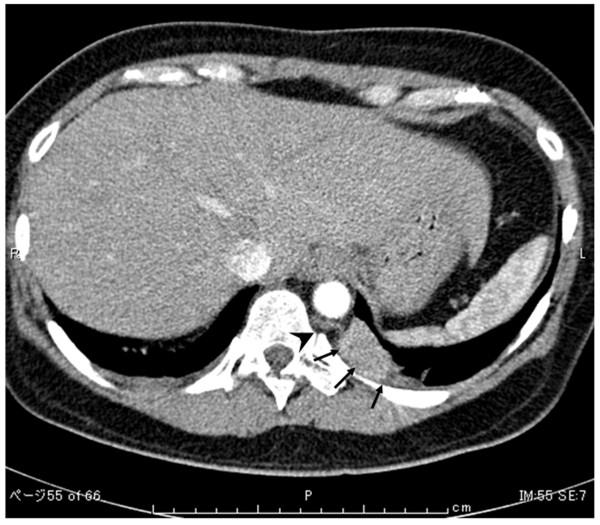
**Preoperative chest computed tomography (CT) scan demonstrated a homogenous, sharply-circumscribed mass in the left lower lobe (*arrow*), and an aberrant feeder artery originating from the descending aorta (*arrowhead*).**

**Figure 2:**
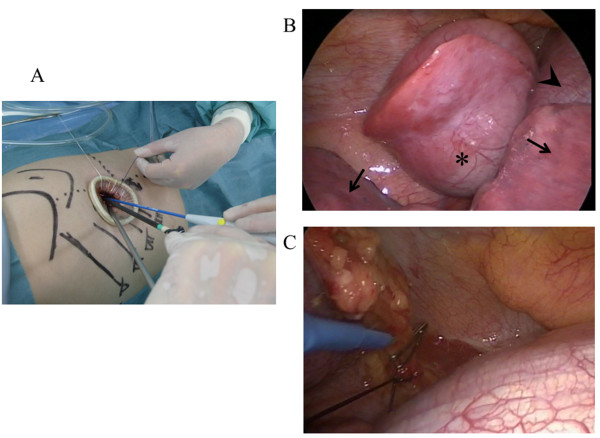
**Intraoperative findings. A**: A 2.5 cm skin incision was made, and a wound retraction system (Alexis Wound Retractor, Applied Medical, Rancho Santa Margarita, CA USA) was then placed through the incision. **B**: A pedunculated tumor was observed, which protruded into the thoracic cavity from the descending aorta. Sequestration lobe (*asterisk*), normal lung (*arrow*) and diaphragm (*arrowhead*). **C**: The aberrant artery was ligated and clipped.

A 45-year-old Japanese woman was admitted to our hospital for further examination of an abnormal shadow found on a chest computed tomography (CT) scan, which indicated a homogenous, sharply-circumscribed mass in the posterior mediastinum. An aberrant feeder artery arose from the descending aorta into the lesion (Figure 1). On the basis of these findings, the patient was diagnosed with extralobar pulmonary sequestration or a solitary fibrous tumor and surgical exploration, and treatment using video - assisted thoracoscopic surgery (VATS) was planned. The patient was administered general anesthesia using one-lung ventilation and was placed in a full right lateral decubitus position. Exploration through the left 7th intercostal space using a 5-mm trocar was performed, and a pedunculated tumor that protruded into the thoracic cavity from the descending aorta was observed (Figure 2B). We determined that single-port surgery was feasible for this type of lesion; therefore, the 5-mm incision was extended to 2.5 cm, and a wound retraction system (Alexis Wound Retractor, Applied Medical, Rancho Santa Margarita, CA, USA) was then placed through the incision. A rigid 5-mm 30° video-thoracoscope, an endograsper and an electric cautery were passed within the same single small incision (Figure 2A). The tumor was suspended using articulating endograspers (Covidien, Norwalk, CT, USA) and resected after clipping and ligation of the anomalous vessel (Figure 2C). The operation time was 45 minutes. Microscopic examination revealed that the vessel had elastic properties similar to that of pulmonary arteries, and markedly dilated bronchial structures accompanied by a tortuous artery corresponding to the cystic mass were observed. The patient had an uneventful course and was discharged on postoperative day 5. At 6-months follow-up, a CT scan demonstrated that the normal parenchyma of the left lower lobe was fully preserved.

## Discussion

Pulmonary sequestration is a rare lesion of the lung parenchyma with an unknown etiology that lacks a normal connection with the tracheobronchial tree and has a blood supply directly from the descending aorta. Surgery is the treatment of choice, which is usually performed through a posterolateral thoracotomy [1], and more recently via VATS because it is minimally invasive, and results in less postoperative pain and faster recovery [2]. Therefore, it has become a commonly used technique for thoracic tumor surgeries [3]. In particular, single-port VATS is useful for specific diseases, such as pneumothorax [4]. To the best of our knowledge, the present report is the first on the use of single-port VATS for pulmonary sequestration. Because only one intercostal space is typically involved in a pulmonary sequestration, the possible advantages of SITS include less postoperative pain, fewer postoperative drainage days, shorter hospital stays, and improved cosmesis compared with the conventional three-port VATS. Some authors have reported less postoperative pain and less paresthesia in patients who underwent minor procedures using a single-port approach compared with the classical three-port approach [5,6]. Careful employment is preferred for intrapulmonary lesions because they tend to require more challenging anatomical or near-anatomical resections as opposed to extrapulmonary lesions.

There are obvious technical problems with SITS. It is not a naturally ergonomic procedure, because the traditional thoracoscopic principles of triangulation are omitted. In addition, positioning of multiple devices through a single small incision in the chest poses problems because instruments can often interfere with each other in the pleural as well as extrapleural spaces, where attachments, such as a camera light can often impede movement. Therefore, to overcome these limitations, the development of new instruments is needed. For example, increasing the length of the camera shaft will allow an assistant to assume a position that did not interfere with that of the surgeon. Furtheremore, the use of a roticulating endograsper aids in achieving triangulation, is compatible with the use of other devices, and can achieve good results.

In the present case, the tumor was resected through a 2.5 cm incision, even though it was 4.2 cm in diameter. A wound retraction system should provide for wound dilation and protection when a specimen requires removal through a small incision. However when the diagnosis is suspected preoperatively, using single-port VATS can avoid obvious excessive invasion.

In conclusion, we recommend minimally invasive single-port VATS for resecting an extralobar sequestration.

## Conclusion

We believe that single-port VATS is a great alternative to the traditional three-port VATS for extralobar sequestration.

## Consent

Written informed consent was obtained from the patient for publication of this case report and any accompanying images. A copy of the written consent is available for review by the Editor- in- chief of this journal.

## Abbreviations

VATS: Video- assisted thoracoscopic surgery; SITS: Single-incision thoracoscopic surgery.

## Competing interests

The authors declare that they have no competing interests.

## Authors’ contributions

YS analyzed and interpreted the patient data. MT performed the literature review, and was a major contributor in writing the manuscript. YH performed the final editing of the manuscript. All authors read and approved the final manuscript.
